# Secondary basal cell carcinoma of scalp after radiotherapy: A case report

**DOI:** 10.1097/MD.0000000000012170

**Published:** 2018-09-07

**Authors:** Frank Chen, Sheau-Fang Yang, Chien-Hung Chen, Ann-Shung Lieu, Shih-Tsung Cheng, Ming-Yii Huang, Hsin-Hua Lee

**Affiliations:** a Department of Radiation Oncology; b Department of Pathology, Kaohsiung Medical University Hospital; c Faculty of Medicine; d Graduate Institute of Medicine, College of Medicine, Kaohsiung Medical University; e Department of Surgery; f Department of Dermatology, Kaohsiung Medical University Hospital, Kaohsiung, Taiwan.

**Keywords:** astrocytoma, BCC, CNS tumor, radiotherapy, secondary cancer

## Abstract

**Rationale:**

Radiotherapy (RT) is widely used for both malignant and benign tumors in order to reduce the risk of recurrence, to promote tumor control, and to improve survival. However, there have been studies reported that RT is also a risk factor of secondary cancer. Very few cases of secondary malignancy after RT to high grade brain cancer have been reported due to short survival of this disease, and most RT-induced malignancies presented with sarcomatous histology. Here we present a patient with basal cell carcinoma (BCC) 14 years after RT to his brain.

**Patient concerns:**

A 28-year-old man without any underlying disease had suffered from left side weakness and clonic-tonic seizures for 12 days.

**Diagnoses:**

His brain images showed a tumor in the right frontal lobe. The pathologic report confirmed anaplastic astrocytoma (WHO Grade III).

**Interventions:**

After craniotomy and tumor biopsy, RT was delivered. Fourteen years later, a gray-colored skin papule was noted in the previously irradiated area. The scalp biopsy revealed BCC. The scalp BCC was adequately resected. He then suffered from brain tumor recurrence and received further craniotomy for three times combined with chemotherapy with temozolomide.

**Outcomes:**

After treatment, follow-up brain images showed that the disease was under control. There was no neurological sequela. For scalp BCC, no skin tumor recurrence has been noted to date after the resection 14 years after initial RT. He has survived for more than 26 years since his initial diagnosis of anaplastic astrocytoma, and more than 12 years from the diagnosis of scalp BCC.

**Lessons:**

Notwithstanding the risk of radiation-induced skin cancer, RT contributed to this patient's survival. The possible late adverse events should be informed to the patients.

## 1 Introduction

Radiotherapy (RT) is widely used for both malignant and benign tumors in order to reduce the risk of recurrence, to promote tumor control, and to improve survival. With improved survival in recent years, the long-term risks from RT, such as developing a second cancer, become more important.^[1]^ Radiation causes DNA damage in both the tumor cells and the surrounding normal cells, which is responsible for this carcinogenetic effect. As a result, some may describe RT as a “double-edged sword,” because while it is a major modality for the treatment of cancer, it can paradoxically also be the cause of cancer.^[2]^ According to a cohort study in 2011 from the US Surveillance Epidemiology and End Results cancer registries, cancer survivors have an approximately 14% higher rate of cancer compared with the general population, and about 8% of second solid cancers might be related to RT for the first cancer.^[1]^ Another cohort study of Hiroshima and Nagasaki atomic bomb survivors by Preston et al reported that the excess risks for all solid cancers as a group and many individual sites exhibit significant variation with gender, attained age, and age at exposure. It was estimated that, at age 70 after exposure at age 30, solid cancer rates increase by about 35% per gray (Gy) (90% CI 28%; 43%) for men and 58% per Gy (43%; 69%) for women.^[3]^

There are some definitions for RT-induced secondary malignancy. According to Cahan's criteria, a radiation-induced malignancy must have arisen in an irradiated field, a sufficient latent period preferably longer than 4 years which have elapsed between the initial irradiation and the alleged induced malignancy, the treated tumor must have been biopsied, the 2 tumors must be of different histology, and the tissue in which the alleged induced tumor arose must have been normal before radiation exposure.^[4]^ The first evidence for carcinogenic potential of ionizing radiation was based on a case report in 1902, which described the development of nonmelanoma skin cancers on the hands of radiation workers.^[5]^ Since then, there are reports for secondary cancer not only at skin but at other organs. Most of our understanding of radiation effects on humans is largely based on the incidence and cancer mortality from Japanese atomic bomb survivors with leukemia and solid tumors.^[6]^ The evidence of radiation-induced skin cancer has been reported in uranium miners, radium dial painters, radiologists, and the patients using early World War II-era high voltage cathode ray tube oscilloscopes, and in the patients treated with X-ray for childhood acne, tinea capitis or thymic enlargement.^[6,7]^ The possible mechanisms underlying the pathogenesis of radiation associated basal cell carcinoma (BCC) development include radiation damaging DNA and the subsequent complex cellular responses, cell signaling pathways that are involved in the chronological progression of radiation-induced tumor lesions, and other factors that can modify susceptibility to radiation-induced BCCs.^[5]^

Few cases of secondary malignancy after RT to high grade brain cancer have been reported due to relatively short survival of this disease, and RT-induced secondary cancer is generally characterized by sarcomatous histology.^[8]^ Here we present a patient with BCC at the scalp who had received brain RT 14 years before the occurrence of BCC.

## 2 Method

The patient provided written informed consent for publication of this report and all accompanying images and tables.

## 3 Case report

A 28-year-old man without any underlying diseases had suffered from left side weakness and clonic–tonic seizures for 12 days. Computed tomography (CT) of the patient's head revealed a hypodensed mass with the size of 3 × 3 × 2.4 cm of the right temporal lobe. He was then admitted, and the magnetic resonance imaging (MRI) of his brain showed cystic astrocytoma in the right parietal lobe without midline shifting. A craniotomy was performed, and the pathology showed astrocytoma (World Health Organization (WHO) grade III). After operation, he received 2-dimensional conformal RT with a total dose of 45 Gy in 25 fractions to the whole brain. Mild skin erythematous change was noted after the completion of RT.

**Figure 1:**
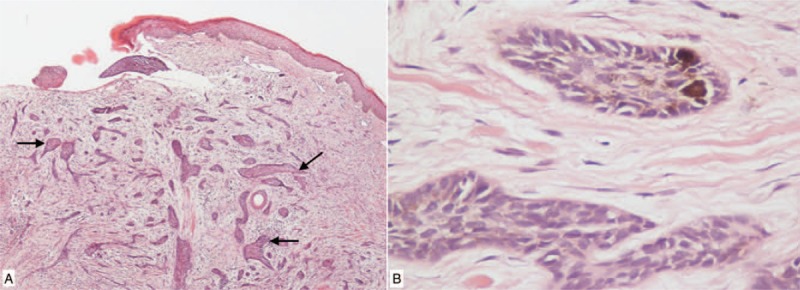
Pathologic images of scalp BCC. (A) Microscopy shows proliferation of nests or cords of basaloid cells with extension into the dermis as arrows indicated (hematoxylin and eosin; original magnification ×40). (B) High power magnification shows basaloid cells bearing elongated nucleus and little cytoplasm with peripheral palisading (hematoxylin and eosin; original magnification ×100).

During a regular follow-up of 14 years after postoperative adjuvant RT, a small red nodule was noted at the patient's right scalp. The size of the nodule increased overtime and the color eventually turned to black within a year. This gray-colored skin papule with a diameter of 2.5 cm has an ulcerative center. He received skin tumor excision at the age of 42. The skin tumor was totally removed with an adequate margin. The pathology revealed BCC. The morphology of the tumor featured basaloid cells which extended into the dermis with elongated nuclei and little cytoplasm. The peripheral cell layer of the tumor masses showed a palisade arrangement of the nuclei (Fig. 1). No skin tumor recurrence has been noted to date.

Two years later, the patient developed headache, left side weakness with unsteady gait at the age of 44. His brain MRI revealed a huge cystic lesion with fluid-fluid level in the patient's right frontal lobe. There were hemorrhage, perifocal edema, mass effect causing mild midline shift. Second craniotomy was performed, and there was presence of microvascular of festoons gliosis, microcalcification and tiny foci of necrosis in the specimen, which favored the diagnosis of glioblastoma (WHO grade IV). He received chemotherapy with temozolomide.

**Figure 2:**
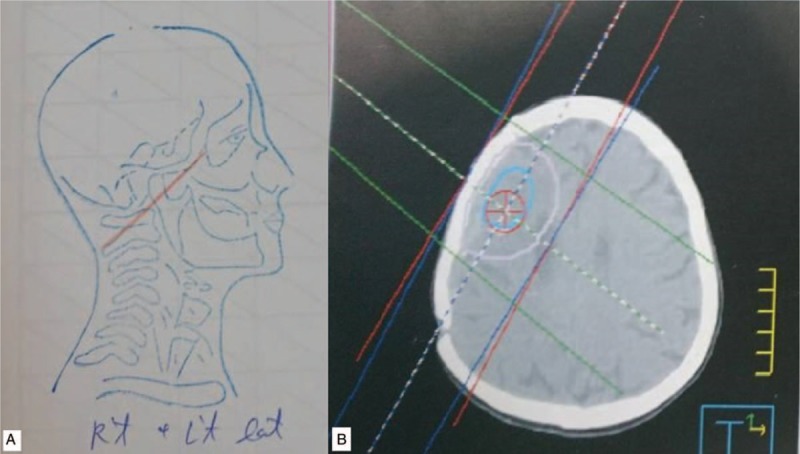
Field arrangement of RT. (A) Field design of the initial 2D-CRT at the age of 28. (B) 3D-CRT treatment plan for recurrent brain cancer at the age of 48.

**Figure 3:**
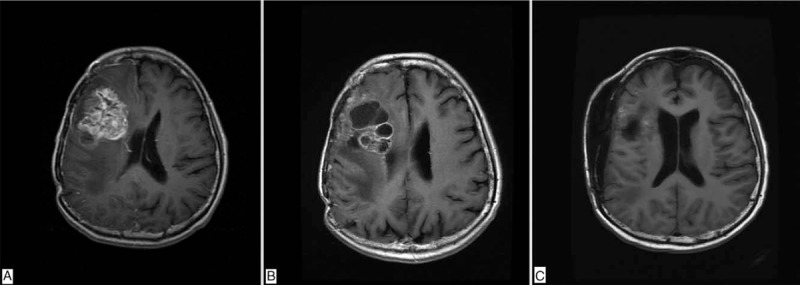
Serial images of brain MRI. (A) Tumor recurrence 20 years after first surgery and RT. (B) Fourth tumor recurrence 25 years after initial diagnosis. (C) Latest MRI of the brain showed that the disease is under control after fourth surgery.

Four years later, there was recurrent astrocytoma in the right frontal lobe with calcification with worsening perifocal edema and mass effect in the follow-up MRI of his brain. Third craniotomy was performed at the age of 48. The pathology revealed anaplastic oligodendroglioma (WHO grade III), as the morphology of tumor showed moderate to high cellularity with dense network of branching capillaries and microvascular proliferation. Adjuvant 3-dimensional conformal RT (3D-CRT) to brain tumor surgical bed was performed with 50 Gy in 25 fractions (Fig. 2). After following for 5 years, MRI of brain showed worsening recurrent tumor in the right frontal lobe and the body of right caudate nucleus. Fourth craniotomy was performed. The pathology revealed anaplastic oligodendroglioma (WHO grade III). The latest follow-up brain image revealed that the disease is under control (Fig. 3). He receives regular follow-ups at our outpatient department. Currently, he has survived for more than 26 years since his initial diagnosis of anaplastic astrocytoma, and more than 12 years from the diagnosis of skin BCC.

## 4 Discussion

The carcinogenesis of RT is a well-known and disturbing adverse late effect. There are reports of intervals of up to 64 years between the exposure to radiation and the emergence of second cancer, suggesting that the risk of carcinogenesis on the irradiated skin persists throughout the patient's entire life.^[9]^ Although radiation exposure is a well-established risk factor for developing secondary malignancy; however, estimating the true incidence of RT-induced secondary malignancy is difficult, for there are many other risk factors that are related to cancer formation.^[10]^ The diagnosis of RT-induced malignancy should be made under strict consideration and other possible risk factors must be excluded. Table 1 summarizes 94 patients with BCC after RT reported before 2017.

**Table 1:**
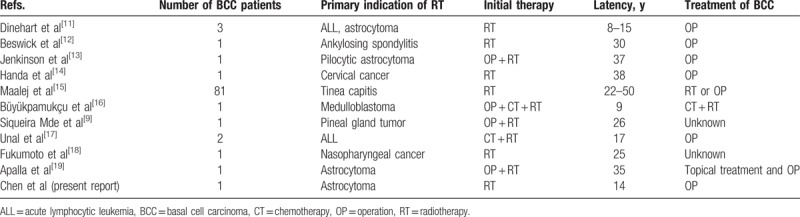
Literature review of RT-induced BCC.

The pathology of RT-induced BCC had been investigated in 1977. There was some difference between electron microscopic pictures of sunlight-induced and X-ray-induced BCC. The frequency of multiple lesions was greater in those induced by X-ray than in the solar or nevoid type. The recurrence and major surgical sequelae appeared to be greater with X-ray-induced lesions.^[20]^ However, the findings of demoscopy and gene expression between BCC in irradiated patients and BCC in nonirradiated patients showed no obvious difference.^[9,21]^ The treatment for RT-induced BCC tends to be more intense for it is prone to recurrence. Besides surgical excision, there are some target therapies approved for the treatment of such aggressive BCC.^[5]^

In our present case, the secondary BCC was treated by surgical excision alone without recurrence to date. The patient suffered from high grade brain cancer initially, and the fact that he successfully survived for so long enabled us to observe the formation of possible RT-induced secondary malignancy. Despite a standard of care combining surgery, RT, and temozolomide chemotherapy, the average overall survival of glioblastoma patients is only 15 months, and even far lower when the patient cannot benefit from this combination. In the United States, the median survival for patients with grade III astrocytoma increased from 15 months in 1999 to 2001 to 24 months in 2008 to 2010 periods.^[22]^ The present case survived for more than 26 years while the median survival for patients with grade III tumors is up to 3 years.^[23]^ Our patient received only 45 Gy to his grade III astrocytoma 26 years ago. Nowadays we prescribed 54 to 60 Gy.

The patient received conventional 2-dimensional conformal RT (2D-CRT) after the first brain tumor removal. Recently, with the transition from 2D-CRT to 3D-CRT, the high dose can be more confined to tumor or surgical bed. Modern RT reduces the volume of normal tissues exposure, which might subsequently lower the risk of RT-induced secondary malignancy. Nowadays, intensity-modulated RT (IMRT) has been more commonly used for its better dose distribution and the decreased high dose to normal tissue. However, the move from 3D-CRT to IMRT involves more fields, which causes a larger volume of normal tissue exposed to lower doses, and that the total body exposure might increase due to leakage radiation from increased monitor units. According to a report by Hall and Wuu,^[2]^ IMRT is likely to almost double the incidence of second malignancies compared with conventional RT from about 1% to 1.75% for patients surviving 10 years, and the numbers may be larger for longer survival, such as in young patients. Kry et al^[24]^ investigated the calculated risk of fatal secondary malignancies from IMRT in patients with prostate cancer, and the data revealed the conservative maximum risk of fatal second malignancy was 1.7% for conventional radiation, 2.1% for IMRT using 10-MV X-rays, and 5.1% for IMRT using 18-MV X-rays.

In recent years, proton therapy has attracted more and more attention for its ideal physical property of Bragg peak. Hall et al^[25]^ suggested that the proton machine employs a pencil scanning beam for reducing low dose volume. Fontenot et al constructed a proton therapy plan and a 6-MV IMRT plan for 3 patients with early prostate cancer. His measured data showed that proton therapy reduced the risk of a secondary malignancy by 26% to 39% compared with IMRT.^[26]^ However, many proton facilities use scattering foil to produce a field of sufficient size, which might produce neutrons, and subsequently result in an effective dose to the patient higher than that characteristic of IMRT. Thus the author concluded that the benefit of protons is only achieved if a scanning beam is used in which the doses are 10 times lower than with IMRT.^[25]^

There are some limitations in our report. First, the initial brain image 26 years ago and clinical pictures of scalp BCC are not available. Second, the diagnosis of RT-induced secondary malignancy is hard to confirm, because it was made by indirect evidence. Third, there was no computerized RT dose distribution in our institute when the patient first received RT 26 years ago, so we cannot evaluate the exact dose at the patient's scalp.

Radiation-induced cancer was primarily defined as a tumor occurring in a radiation field, which has the pathological features different from those of the primary cancer, and a period that was long enough to develop a cancer. There is still no consistent definition of the time interval, but we believe that 14 years could satisfy the criterion. Although the increased risk of malignant cell transformation after RT has been revealed for decades, most cancer patients still receive their necessary treatment under most circumstances as we weigh the benefit against the carcinogenic complication. Ironically, the limited life expectancy for our most cancer patients just saves us from the embarrassing misfortune.

## 5 Conclusion

Notwithstanding the risk of radiation-induced skin cancer, RT contributed to this patient's survival. The possible late adverse events should be informed to the patients.

## Acknowledgments

The authors thank the help from Alexander Gittin in the Department of Post-Baccalaureate Medicine, College of Medicine, Kaohsiung Medical University. (Mr. Gittin has given permission to be named.)

## Author contributions

**Conceptualization:** Hsin-Hua Lee.

**Data curation:** Frank Chen, Sheau-Fang Yang, Chien-Hung Chen, Hsin-Hua Lee.

**Formal analysis:** Frank Chen.

**Funding acquisition:** Hsin-Hua Lee.

**Resources:** Frank Chen, Ming-Yii Huang, Hsin-Hua Lee.

**Supervision:** Sheau-Fang Yang, Ann-Shung Lieu, Shih-Tsung Cheng, Ming-Yii Huang, Hsin-Hua Lee.

**Validation:** Ann-Shung Lieu, Shih-Tsung Cheng.

**Writing—original draft:** Frank Chen.

**Writing—review & editing:** Sheau-Fang Yang, Hsin-Hua Lee.
